# Effectiveness of Yoga for Hypertension: Systematic Review and Meta-Analysis

**DOI:** 10.1155/2013/649836

**Published:** 2013-05-28

**Authors:** Marshall Hagins, Rebecca States, Terry Selfe, Kim Innes

**Affiliations:** ^1^Department of Physical Therapy, Long Island University, Brooklyn Campus, One University Plaza, Brooklyn, NY 10021, USA; ^2^Department of Epidemiology, West Virginia University School of Public Health, Morgantown, WV 26506-9190, USA; ^3^Center for the Study of Complementary and Alternative Therapies, University of Virginia Health System, Charlottesville, VA 22908-0782, USA; ^4^Department of Physical Medicine and Rehabilitation, Center for the Study of Complementary and Alternative Therapies, University of Virginia Health System, Charlottesville, VA 22908-0782, USA

## Abstract

*Objectives*. To systematically review and meta-analyze the effectiveness of yoga for reducing blood pressure in adults with hypertension and to assess the modifying influences of type and length of yoga intervention and type of comparison group. *Methods*. Academic Search Premier, AltHealthWatch, BIOSIS/Biological Abstracts, CINAHL, Cochrane Library, Embase, MEDLINE, PsycINFO, PsycARTICLES, Natural Standard, and Web of Science databases were screened for controlled studies from 1966 to March 2013. Two authors independently assessed risk of bias using the Cochrane Risk of Bias Tool. *Results*. All 17 studies included in the review had unclear or high risk of bias. Yoga had a modest but significant effect on systolic blood pressure (SBP) (−4.17 [−6.35, −1.99], *P* = 0.0002) and diastolic blood pressure (DBP) (−3.62 [−4.92, −1.60], *P* = 0.0001). Subgroup analyses demonstrated significant reductions in blood pressure for (1) interventions incorporating 3 basic elements of yoga practice (postures, meditation, and breathing) (SBP: −8.17 mmHg [−12.45, −3.89]; DBP: −6.14 mmHg [−9.39, −2.89]) but not for more limited yoga interventions; (2) yoga compared to no treatment (SBP: −7.96 mmHg [−10.65, −5.27]) but not for exercise. *Conclusion*. Yoga can be preliminarily recommended as an effective intervention for reducing blood pressure. Additional rigorous controlled trials are warranted to further investigate the potential benefits of yoga.

## 1. Introduction

Current estimates suggest that over 76 million US adults suffer from hypertension [1] and that blood pressure is well controlled in less than 50% of these individuals [2]. Uncontrolled hypertension is thought to be responsible for 62% of cerebrovascular disease and 49% of ischemic heart disease [3] and is estimated to cost the United States $93.5 billion in health care services, medications, and missed days of work in 2010 [4]. The cost of drugs, drug interactions, and nonadherence with the drug regimen all contribute to current high rates of uncontrolled hypertension. Alternative, less expensive methods to reduce blood pressure that have lower risk of drug interactions and which may convey the benefits of long-term adherence are much needed. 

Yoga is one such alternative healthcare practice thought to improve blood pressure control [5–7]. There is no single definition of the practice of yoga, that is universally accepted although it is generally described as an ancient tradition (originating 5,000 to 8,000 years ago) [8–10] that incorporates postures, breath control, and meditation, as well as specific ethical practices [11, 12]. The number of yoga practitioners continues to rise, with current estimates indicating at least 15.8 million people in the United States (6.9% of Americans) practice yoga [13]. Most relevant to the issue of blood pressure control is that yoga is increasingly being suggested by American health care providers as a means of enhancing health [13]. Of the many benefits ascribed to yoga practice, blood pressure control is among the most studied [7]. While several reviews regarding the potential benefits of yoga for reducing blood pressure and other cardiovascular disease risk factors have been published [5, 7, 14–17], most have stated that the quality of the studies are generally poor. Additionally, few reviews have specifically focused on blood pressure control, and meta-analyses are lacking. Thus, the degree to which yoga may decrease blood pressure as well as the potential modifying effects of type of yoga intervention and type of comparison group remain unclear. To address these gaps, this paper presents a systematic review and meta-analysis of controlled studies (randomized and nonrandomized) examining the effects of yoga practice on systolic and diastolic blood pressure in individuals with prehypertension or hypertension.

## 2. Methods

### 2.1. Literature Search

Methods of the analysis and inclusion criteria were specified in advance but were not documented in a publicly available protocol. A systematic literature search was carried out using the databases Academic Search Premier, AltHealthWatch, Biosis/Biological Abstracts, CINAHL Plus with Full Text, Cochrane Library, Embase, MEDLINE, PsycINFO, PsycARTICLES, Natural Standard, and Web of Science. Additional studies were identified by searching bibliographies of reviews, all studies included in this review, and select uncontrolled studies of yoga and blood pressure. Search terms included yoga or yogi* or yama or niyama or pratyaharaor dharana or dhyana or samadhi or asana combined with “blood pressure” or hypertension or hypertensive or systolic or diastolic. 

### 2.2. Inclusion/Exclusion Criteria

1. *Types of studies*: Peer reviewed, English language, controlled studies (either a randomized controlled trial (RCT) or a non-RCT) published between January 1966 and March 2013 were included. Cross-sectional studies, case series, dissertations, and abstracts/posters were not included.
2. *Participants*: Adults (mean age ≥ 18 years) with prehypertension or hypertension.
3. *Interventions*: Given the large variability in practices associated with the term “yoga,” only papers that explicitly labeled the intervention as “yoga” were examined. Consequently, we excluded studies that reported on the effects of any form of meditation, mindfulness-based stress reduction, or relaxation response which the authors did not specifically label as yoga. Studies of Transcendental Meditation (TM), a form of yogic meditation, were excluded, since a comprehensive review and meta-analysis regarding the effects of TM on blood pressure has been recently conducted [18]. In addition, we excluded studies only examining immediate changes following a single yoga session. We also excluded studies examining only practices rarely performed currently but historically associated with yoga such as bloodletting, starvation, and cleansing of the stomach.
4. *Outcome measures*: Blood pressure (mmHg) was the only outcome of interest (systolic and diastolic). Studies which did not provide blood pressure data (effect size and/or variability estimates) were excluded.

### 2.3. Data Extraction

Abstracts were initially examined by a single investigator (MH). Independent extraction of data on potentially eligible articles was performed by two authors (MH/RS) using predefined data fields. Disagreements between reviewers were resolved by discussion to achieve consensus. Blood pressure values with standard deviation or standard error as well as participant health status, type of yoga intervention, type of comparison group, demographic characteristics, number of participants enrolled and completing the study, location of the study, reporting of adverse events, and methods for measurement of blood pressure were gathered from each paper. Systolic and diastolic blood pressures (mmHg) were the only measures of treatment effect investigated by meta-analysis. Mean posttest values, or change scores when available, were used for analysis. Where no standard deviations were available they were calculated from the standard error. For otherwise eligible studies that did not provide blood pressure values, corresponding authors were contacted by email in an effort to obtain the information needed for inclusion in this review.

### 2.4. Risk of Bias

The risk of bias for each study was determined independently, but unblinded, by the same two authors using the criteria of the Cochrane Risk of Bias Tool. Disagreements were resolved by discussion to achieve consensus [36]. Studies which had unclear or high risk of bias in one or more key domains (selection, detection, attrition, reporting but not *performance* bias) were considered at high risk of bias. 

### 2.5. Data Analysis/Assessment of Heterogeneity

Reference Manager (RevMan) Version 5.1 from the Cochrane Collaboration [37] was used to analyze all data and construct forest plots, as well as to evaluate heterogeneity across studies and to perform sensitivity and subgroup analyses. Statistical heterogeneity across studies was tested using Tau^2^, Chi^2^, and the method proposed by Higgins and Thompson [38]. Given the broad nature of the research question and the variability within the target studies by type of yoga and type of comparison group we expected a large degree of heterogeneity. Consequently we planned use of a random effects model for all comparisons [39]. 

### 2.6. Subgroup and Sensitivity Analysis

A primary methodological concern was whether controlled but nonrandomized studies should be included in the meta-analysis given that such studies by definition suffer from selection bias. Consequently, sensitivity analyses were conducted to assess potential variation by presence or absence of random participant allocation. In an effort to be maximally inclusive of relevant data we included studies whose populations were not explicitly hypertensive but was composed of individuals with cardiac health related issues (e.g., diabetes, metabolic syndrome) with a majority of the study participants currently hypertensive. Consequently, sensitivity analyses were conducted to assess potential variation by presence or absence of study inclusion criteria that required participants to be hypertensive. 

We hypothesized a priori that variation in intervention practices would likely contribute substantial heterogeneity to the outcomes. Consequently, subgroup analyses were performed based on duration of the yoga intervention and on yoga practice components included in the intervention Yoga interventions were divided into 3 categories: (1) those that incorporated postures, meditation, and breathing (“3-element yoga”); (2) those that included fewer than the 3 yoga practices just described; (3) yoga using any combination of the three elements plus one or more additional intervention(s). We also categorized yoga intervention by total time of practice, distinguishing between studies where total time of practice was shorter or longer than the mean duration across all studies. Finally, we performed a subgroup analysis based on comparison group, as we expected between-group effects to vary depending on the control condition. For this subgroup analysis, we used three categories of comparison groups: (1) usual care, no treatment, or wait list; (2) exercise; and (3) attention control or active, nonexercise comparator.

## 3. Results

### 3.1. Literature Search

**Figure 1:**
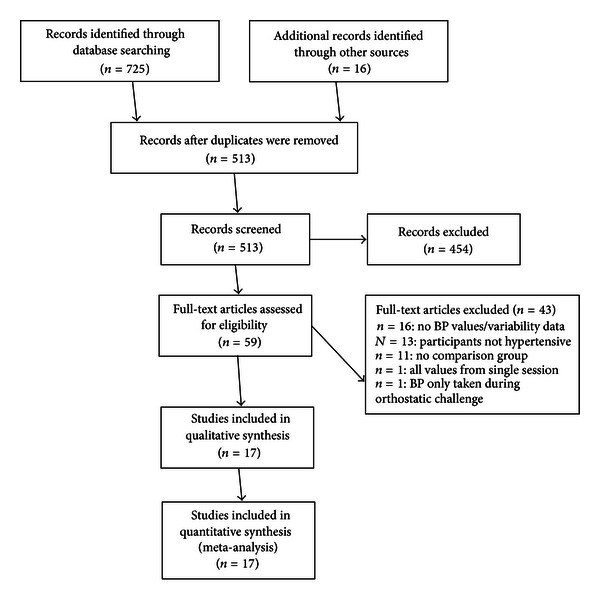
Flow Diagram of article selection.

The initial database searching located 725 potentially eligible articles; an additional 16 papers were identified through other sources, bringing the total number of articles for preliminary review to 741 (Figure 1). Of these, 228 were excluded as duplicates, and 454 for failure to meet inclusion criteria after review of the abstract. Of the remaining 59 articles a full text review yielded 16 studies meeting our full eligibility criteria. An additional 15 studies did not report blood pressure values or variability data, but met all remaining eligibility requirements [32, 36, 40–52]; the primary authors of these studies were contacted to request data. Only one author agreed to provide data and this study [32] was included in the analysis, bringing the total eligible articles to 17. Several papers examined more than one comparison group. These studies were considered independent trials [53] and consequently 22 trials within the 17 studies were identified for analysis.

### 3.2. Study Characteristics

Characteristics of each study are detailed in Table 1. Most studies were conducted in India (*n* = 8) and the USA (*n* = 6), with the remaining conducted in The Netherlands (*n* = 1), Brazil (*n* = 1), and Thailand (*n* = 1). The total number of enrolled participants examined across all included studies was 1013, with 473 (46.7%) assigned to the yoga group and 540 (53.3%) assigned to the comparison group. The total number of participants completing the studies was 943 (yoga = 427; controls = 516) with mean drop-out rates of 9.7% and 4.4% for the yoga and comparison groups, respectively. Of the studies that reported gender (*n* = 14), approximately 38% of study participants were male (some studies reported percentages and did not clarify if gender applied to enrolled participants or to those completing the study). The mean study sample size (using number of participants who completed the study) was 55.4 (±31.8), ranging from 20 to 120 participants. Ten (58.8%) of the 22 studies incorporated three elements of yoga (postures, meditation, and breathing) with no additional interventions, while 4 (23.5%) used two or fewer of the elements, and 3 (17.6%) used various elements of yoga in combination with additional interventions. Within the 22 trials three categories of comparison groups were identified: 13 (59%) no treatment or usual care; 3 (13.6%) exercise; 5 (22.7%) various types of nonyoga, nonexercise interventions. Potential adverse events were not reported in 12 (70.1%) of the studies, the absence of adverse events were reported in 4 (23.5%) of the studies, and one study (5.8%) [21] reported three adverse events within the yoga group. The mean length of time used for yoga practice was 58.9 (±56.1) hours; 12 studies had fewer hours and 5 had more hours than the average.

#### 3.2.1. Risk of Bias

**Figure 2:**
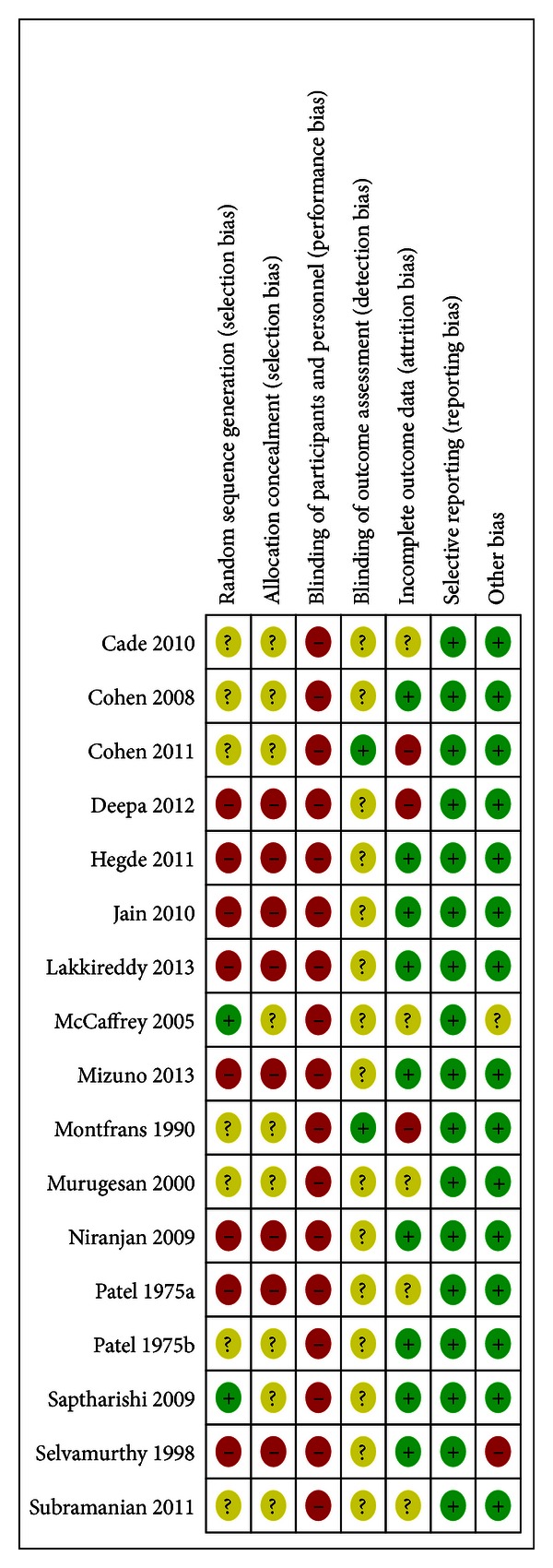
Risk of bias summary.

Categorization of the risk of bias at the individual study level is presented in Figure 2. No studies achieved a low risk of bias as all had an unclear or high risk of bias within at least one major domain. *Sequence Generation and Treatment Allocation: *15 of the 17 studies had unclear or high risk of selection bias as 8 of the studies were nonrandomized and 7 failed to describe sequence generation or allocation. *Blinding of participants: *all studies had high risk of bias for blinding of intervention. Due to the required participatory nature of yoga this category was not considered a primary domain for risk of bias. *Blinding of outcome assessors:* all studies had an unclear risk of bias for outcome assessment with the exception of two which reported blinding (low risk of bias) [21, 23]. *Attrition bias* varied across groups. Eight of 17 studies were assigned unclear or high risk of attrition bias as 3 [21, 23, 28] had high drop-out rates and/or no report of intention-to-treat analysis (high risk of bias) and in 5 studies the drop-out rates exceeded 15%, but were comparable between groups (unclear risk of bias). In the remaining studies (*n* = 9) both intervention and comparison groups had dropout rates of 15% or less or conducted an intention to treat analysis (low risk of bias); and *Selective reporting*: as only one outcome (blood pressure) was examined within this review and studies were only included if these values were described in the report. *Other bias: *all studies had low risk of other biases except one assigned high risk of bias as baseline values differed significantly between groups [35] and one assigned unclear risk of bias as [22] as values were inconsistent between text and tables.

#### 3.2.2. Effects of Yoga on Blood Pressure

**Figure 3:**
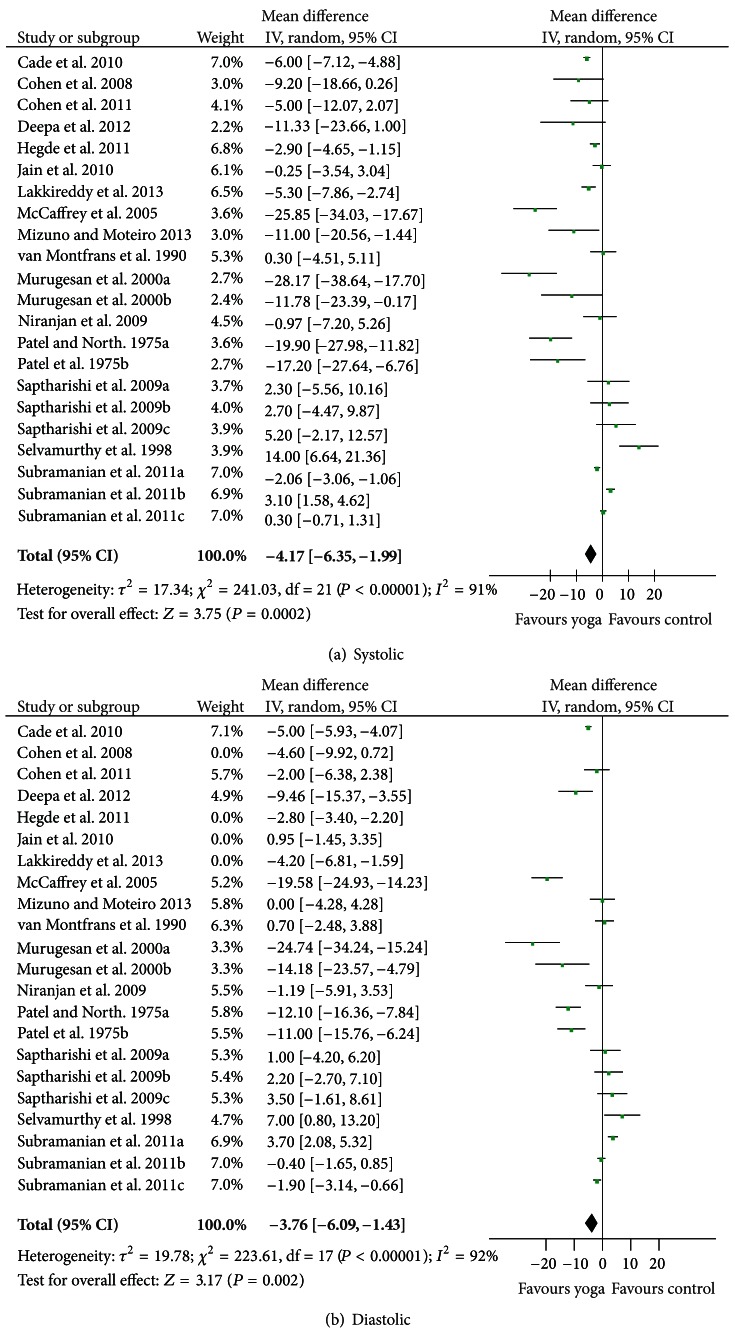
Forest plots of overall effect of yoga on prehypertension and hypertension: (a) systolic, and (b) diastolic.

As illustrated in Figures 3(a) and 3(b), yoga had a modest but significant effect on both systolic (*Z* = 3.75, (*P* = 0.0002); −4.17 mmHg [−6.35, −1.99]) and diastolic blood pressure (*Z* = 3.86, (*P* = 0.0001); −3.26 mmHg [−4.92, −1.60]). There was substantial heterogeneity present across the included studies: Tau^2^ = 17.34; Chi^2^ = 241.03, df = 21, (*P* < 0.00001), *I* ^2^ = 91% for systolic and Tau^2^ = 11.17; Chi^2^ = 234.96, df = 21, (*P* < 0.00001), *I* ^2^ = 91% for diastolic. 

### 3.3. Sensitivity Analysis

Sensitivity analysis was performed by comparing the meta-analysis from all 17 studies with a meta-analysis of the RCTs only (*n* = 9). A second sensitivity analysis was performed by comparing the meta-analysis from all 17 studies with a meta-analysis of the studies which focused on cardiac related health issues but did not have hypertension as an explicit inclusion criteria, although the majority of the participants had hypertension (*n* = 5) [19, 20, 24–26]. For both sensitivity analyses, no substantive differences in either the direction or magnitude of effect size were created by removing the identified studies. Consequently, the findings of all 17 studies were pooled for these analyses.

The number of trials, number of participants, and effect sizes for subgroups is reported in Table 2. Subgroup analyses for systolic and diastolic blood pressure indicated a significant modifying effect of type of yoga intervention (Chi^2^ = 14.30, *P* = 0.0008 and Chi^2^ = 13.14, *P* = 0.001, resp.) and type of comparison group (Chi^2^ = 48.30, *P* = 0.00001 and Chi^2^ = 14.89, *P* = 0.0006, resp.) but not for duration of yoga practice (Chi^2^ = 2.45, *P* = 0.12 and Chi^2^ = 0.61, *P* = 0.43, resp.). The subgroup analysis for type of yoga intervention suggests that incorporating three elements of practice (posture, meditation, and breathing) is associated with significant reductions in blood pressure whereas yoga interventions using two or fewer elements of yoga practice or that combine yoga practice with additional interventions are not (Table 2). The subgroup analysis regarding type of comparison group suggests that RCTs comparing yoga to usual care showed that yoga had a significant effect on blood pressure compared to no treatment but not when compared to exercise or other types of treatment (Table 2).

## 4. Discussion 

When the results of all 17 studies (22 trials) examined in this review are pooled, yoga was associated with a small but significant decline in both systolic and diastolic blood pressure (−4.17 and −3.26 mmHg, resp.). Further, yoga's effects on blood pressure varied by type of yoga intervention and by comparison group, but not by duration of yoga practice. These subgroup differences may partially explain the high degree of heterogeneity found across all studies. The level of overall blood pressure reduction achieved by yoga is similar to that of other lifestyle modifications advocated by current guidelines, including exercise [27] and reduced intake of sodium and alcohol [3]. While the overall declines resulting from yoga practice were modest, even small reductions in blood pressure have been shown to reduce risk for coronary heart disease and stroke [29, 30].

When the analysis was restricted to studies using interventions incorporating three elements of yoga practice (postures, meditation, and breathing), larger reductions of −8.17 (systolic) and −6.14 (diastolic) mmHg were observed. Declines of this magnitude are of clear clinical and prognostic significance [3]. To our knowledge, this is the first study to provide preliminary evidence supporting increases in blood pressure reduction associated with specific methods of yogic practice.

Yoga was also associated with a significant decline in systolic (−7.96 mmHg) and diastolic blood pressure (−5.52 mmHg) relative to no treatment, but not when compared to exercise or other types of interventions. It is well known that exercise and some of the other active interventions used within the included studies decrease blood pressure relative to no treatment [27, 29] in the range of 3–9 mmHg (systolic). Given that their effects are comparable in magnitude and direction to those observed with yoga, it is not surprising that we found no significant benefit of yoga when it was compared to an alternate active treatment. 

### 4.1. External and Internal Validity

The participants of studies included in this report were male and female adults with prehypertension or hypertension with or without cardiovascular disease. The findings of this report are thus applicable to the majority of individuals with elevated blood pressure. Most studies assessed gentle yoga programs of relatively short duration that could be readily implemented in this clinical population.

Unfortunately, overall quality of studies included in this meta-analysis was poor. All had either unclear or high risk of bias on one or more primary domains. The most common risk of bias was the failure to blind (or to report blinding of) participants. However, studies requiring active participation in an instructor-led intervention cannot be blinded and consequently we did not consider this a primary domain reflecting study quality. However, only 2 of the 17 studies reported blinding of outcome assessors, an entirely feasible method for active intervention studies. In addition, 8 of 17 studies had high or unclear risk of attrition bias and 15 of 17 studies had high or unclear risk of selection bias.

### 4.2. Strengths and Weaknesses

This is the first meta-analytic review to examine the effects of yoga on blood pressure. Strengths of this study include the systematic literature search using multiple databases and based on criteria defined a priori, assessment of studies by multiple authors, a priori decisions regarding appropriate subgroup analyses, and use of well-established meta-analysis procedures for our analyses. One limitation of the current study is we did not assess other potentially contributing factors such as style of yoga, qualifications of instructors or teaching styles, practice environment, participant characteristics such as physical fitness and yoga experience, as well as blood pressure assessment procedures, and other methodological issues. Additional limitations are the restriction to English-language publications, to the selected database sources, and to studies that reported complete blood pressure values. 

Exclusion of studies that used yogic interventions but did not label the intervention as such may also have introduced bias. Because there are no universally accepted standards for what constitutes yoga practice, reviews such as this one must necessarily create criteria to define yoga for the purposes of analysis. In this review we excluded studies of certain therapies that, while not defined by the authors as “yoga,” could arguably be viewed as yogic practices. These included, for example, studies of certain meditation techniques that, while generally considered yogic practices, were not described as such. Given that there is already considerable evidence suggesting that meditation is effective in lowering blood pressure; [18, 31, 33] exclusion of these studies may have biased our subgroup analysis of effects by yoga program type. Thus, our findings suggesting that programs incorporating three core elements of yoga (postures, meditation, and breath control) led to significant blood pressure reductions while yoga programs using two or less elements of yoga did not lead to significant reductions in blood pressure reduction should be interpreted with caution. In addition, although some studies included in this review were of reasonably long duration (189 hours) [25], the majority of studies (*n* = 10) were less than 50 hours. Future studies should consider methods, as far as are feasible, which more closely resemble suggested yogic practice (many months to years of practice). Given that the studies within this report had substantial potential risk of bias across multiple domains, future studies should focus on the use of well-designed RCTs which blind outcome assessors, use intention to treat analyses, fully report adverse events, and incorporate measures of treatment expectancy.

## 5. Conclusion

The current study is the first meta-analysis to examine the effects of yoga on blood pressure among individuals with prehypertension or hypertension. Overall, yoga was associated with a modest but significant reduction in blood pressure (*≈*4 mmHg, systolic and diastolic) in this population. Subgroup analyses demonstrated larger, more clinically significant reductions in blood pressure for (1) interventions incorporating 3 basic elements of yoga practice (postures, meditation, and breathing) (*≈*8 mmHg, systolic; *≈*6 mmHg, diastolic) but not for more limited yoga interventions; (2) yoga compared to no treatment (*≈*8 mmHg, systolic; 6 mmHg, diastolic) but not compared to exercise. These reductions are of clear clinical significance and suggest that yoga may offer an effective intervention for reducing blood pressure among people with prehypertension or hypertension. As none of the included studies had methodologies with low risk of bias in primary domains additional rigorous controlled trials are warranted to further investigate the potential benefits of yoga for improving blood pressure in these populations and to determine optimal yoga program design and dosing.

## Figures and Tables

**Table 1:** Characteristics of studies (*n* = 17), randomized, nonrandomized controlled trials.

Author/date/ location	Sample size (yoga, control)	% completed (yoga, controls)	Study population (categorization)	Yoga intervention description (categorization)	Comparison group(s) (categorization)	Yoga frequency/duration of session and total sessions	Total time in minutes	BP measure	Adverse events
Randomized controlled trials									

Cade et al. [19] 2010 USA	34, 26	85.3, 80.8	HIV infected adults with moderate CVD risk, 83% with hypertension, 18–70 yrs., 47% male, most on multiple medications related to HIV status and CVD risk including BP meds, unclear control of changes in BP meds during study	P, M, B; Ashtanga Vinyasa; encouraged to practice at least one time per week at home/no homework compliance measures [1]	usual care [1]	2.5 wk/60 mins/20 wks	3000	NR	NR

Cohen et al. [20] 2008 USA	14, 12	85.7, 100	Underactive, overweight adults, with metabolic syndrome, 30–65 yrs., 25% males, 59% on at least one BP med., no reported control for BP meds during study	P, M, B; “Restorative” warm up of stretches and breathing exercises followed by 10 poses. Home practice: 3x week for 30 minutes each/home diary for compliance [1]	No treatment [1]	Intro class 180 mins + 2x wk/90 mins/5 weeks + 1x wk/5 wks + reported mean 117 mins × 10 wks	2700	S	None

Cohen et al. [21] 2011 USA	46, 32	56.5, 96.8	Hypertensive adults, 22–69 yrs., 50% males, none on BP meds by exclusion at recruitment	P, M, B; Iyengar yoga. Home practice during weeks 6–12 one time per day for 25 minutes/home diary for compliance [1]	Enhanced usual care; motivational and behavioral components of life style modifications, for example, reduction of weight and ingestion of sodium and alcohol [3]	2x wk/70 mins/6 wks + 1x wk/6 wks	1260	Am	3 (7%)

McCaffrey et al. [22] 2005 Thailand	32, 29	84.4, 93	Hypertensive adults, age range not reported/mean = 56 yrs., 35% male, none on BP meds by exclusion at recruitment, controlled for those who began BP meds by dropping from study	P, M, B; unspecified type of yoga it appears to be independent practice rather than classes using booklets based on yogic principles for guidance. No information about training in yoga practice. As appears that all practice was at home (no group classes)—no additional home practice [1]	Usual care [1]	3x wk/63 mins/8 wks	1512	NR	NR

van Montfrans et al. [23] 1990 The Netherlands	19, 23	94.7, 73.9	Hypertensive adults, 24–60 yrs., 51% male, none on BP meds by exclusion at recruitment, no reported control for BP meds during study	P, M, B; multimodality program. Hatha yoga plus progressive relaxation and autogenic training for 8 weeks followed by 10 months of independent practice 2x day with cassette tape. All practice was at home except first 8 weeks so no additional home practice [3]	Education about stress and hypertension. Relaxation in comfortable chair [3]	1x wk/60 mins/8 wks plus home practice of 7x/wk/30 mins/40 wks	480	Am	NR

Murugesan et al. [24] 2000 India	11, 11, 11	100, 100, 100*	Hypertensive adults, 35–65 yrs., gender not reported, none on BP meds by exclusion at recruitment, one comparison group used BP meds	P, M, B; unspecified type of yoga. List of asanas provided plus Om recitation and meditation. No home practice [1]	No treatment [1], medication [3]	12x wk/60 mins/11 wks	7920	S	NR

Patel and North [25] 1975 USA	18, 18	94.4, 94.4	Hypertensive adults, 34–75 yrs., 38% male, 94% on BP meds at enrollment, no reported control for BP meds during study	Not reported if P, M, B; multimodality, unspecified type of yoga. Yoga plus education regarding hypertension, “yoga relaxation methods,” “transcendental meditation,” and skin resistance biofeedback. “Instructed to practice relaxation and meditation twice per day.” No homework compliance measures [3]	No treatment [1]	2x wk/30 mins/6 wks	360	S	NR

Saptharishi et al. [26] 2009 India	27, 30, 28, 28	77.8, 96.7, 96.4, 89.3	Young pre- and hypertensive adults, age range not reported/mean of all groups 22 yrs., 67% male, BP meds status not a recruitment criterion and not reported	P, B; unspecified type of yoga; postures and breath practices as per reference to previous paper. It appears that only practice is home practice “encouraged to practice yoga.” No compliance measures reported [2]	No treatment [1] walking program [2], reduction of salt intake [3]	5x wk/45 mins/8 wks	1800	S	NR

Subramanian et al. [27] 2011 India	25, 25, 23, 25	100, 100, 100, 84	Young pre- and hypertensive adults, age range not reported/mean of all groups 23 yrs., 65% male, BP meds status not a recruitment criterion and not reported	P, B; unspecified type of yoga; postures and breath practices as per reference to previous paper. It appears that only practice is home practice “encouraged to practice yoga.” No compliance measures reported [2]	No treatment [1] walking program [2], reduction of salt intake [3]	5x wk/45 mins/8 wks	1800	S	NR

Non randomized controlled trials									

Deepa et al. [28] 2012 India	15, 15	100, 100*	Hypertensive adults, 45–65 yrs., 53% male, 100% on BP medication	P, M, B; Yoga Nidra: it begins with single sitting pose and single breath exercise followed by 45 mins of corpse pose meditation led by instructor. No home practice as this occurred 2x/day [1]	Usual care, in this case, continued medication [1]	10x wk/60 mins/12 wks	7200	S	NR

Hegde et al. [29] 2011 India	60, 63	95, 100	Adults with Type 2 diabetes, 40–75 yrs., gender not reported, BP meds status and recruitment criterion not reported	P; unspecified type of yoga—19 asanas described only. No home practice described [2]	Usual care [1]	Class length and frequency not reported: class sessions occurred over 3 months	NR	NR	None

Jain et al. [30] 2010 India	57, 30	100, 100	Adults, hypertension status not described (although mean BP values suggest pre-hypertension of both groups), yoga group 30–60 yrs., age of control group not reported, 60% male in yoga group, gender not reported in control group, BP meds status and recruitment criterion not reported	P, M; unspecified type of yoga, Surya Namaskar + “Sharir Sanchalan”, and “Bhajan Cassette” No home practice as this occurred daily [2]	No description of any kind for control group [1]	7x wk/90 mins/18 weeks	11340	S	NR

Lakkireddy et al. [31] 2013 USA	52, 49	94, 100	Adults with paroxysmal atrial fibrillation, 39% with known hypertension, (mean BP values across groups suggest pre-hypertension) 18–80 yrs., 47% male, BP meds not a recruitment criteria but reported and controlled for during the interventions	P, M, B: iyengar: home practice encouraged with DVD provided but no compliance measures for homework [1]	Wait list control, same participants for yoga and control group [1]	3x wk (median value)/60 mins/12 wks.	2160	NR	None

Mizuno and Monteiro [32] 2013 Brazil	17, 16	100, 100	Hypertensive adults, age range not reported/mean(SD) yoga group = 67 (7) and control group = 62 (12) yrs., 15% male, majority of participants on blood pressure medication, meds controlled for in study	P, M, B; Unspecified type of yoga, although reference for asanas is Iyengar text; Pranayama, then asana, end with breathing meditation [1]	Usual care [1]	3x wk/90 mins/16 wks	4320	NR	None (PC)

Niranjan et al. [33] 2009 India	16, 16	100, 100	Hypertensive adults, age not reported, gender not reported; BP meds status and recruitment criterion not reported	P, M, B: Unspecificed type of yoga, chanting, prayer, asana, breathing exercises, ending with Savasana. No home practice described [1]	Standard exercise, warm up, stationary bike 30 mins, cool down total = 45 mins; intensity not described [2]	4x wk/60 mins/36 wks	8640	NR	NR

Patel [34] 1975 USA	20, 20	100*	Hypertensive adults, age range not reported/mean = 57 yrs., 31% male, 64% on BP meds at enrollment, no reported control for BP meds during study	Not reported if P, M, B; Multimodality, unspecified type of yoga. Yoga plus “psychophysical relaxation exercise based on yogic principles and reinforced by bio-feedback instruments.” No home practice [3]	No treatment [1]	3*x* wk/30 mins/12 wks	1080	NR	NR

Selvamurthy et al. [35] 1998 India	10, 10	100, 100	Hypertensive adults, 100% male, age range not reported/groups divided by age with mean of yoga 50 yrs. and mean of control group 34 yrs., BP meds gradually withdrawn on all participants prior to study onset	P; Unspecified type of yoga; described several specific asanas. No homework practice [1]	Tilt table [3]	Frequency/time in class not reported. Class sessions occurred over 3 weeks	NR	S	NR

Yoga intervention categorization: P: postures; B: breathing; M: meditation; 1 = P + M + B, 2 = any 2 of these or less; 3 = (±P ±M ±B) ± other interventions.

Comparison group categorization: 1 = no intervention or usual care, 2 = exercise or exercise + additional intervention, 3 = nonexercise intervention.

BP:blood pressure: measurement methods: S: sphygmomanometer; M: machine; Am: ambulatory blood pressure, and NR: not reported.

Males within study based on enrollment data, if not available, data of participants that completed study was used.

Adverse event: NR: not reported; PC: per personal communication with corresponding author.

*Number of participants at completion not reported/estimate assumes 100% completion.

**Table 2:** Results of subgroup analyses: effect sizes, number of trials, and number of participants per subgroup.

Subgroup category	Number of trials	Number of participants	Effect size (confidence interval), mmHg	
			Systolic	Diastolic
Type of yoga intervention*				
(1) P, M, B	11	431	−8.17 (−12.75, − 3.89)	−6.14 (−9.39, − 2.89)
(2) 2 or less of PMB	8	403	0.19 (−1.70, 2.07)	0.38 (−1.55, 2.32)
(3) (±P ± M ± B) + other intervention	3	109	−11.87 (−26.43, 2.70)	−7.35 (−16.20, 1.50)
Type of comparison group*				
(1) No intervention or usual care	13	656	−7.96 (−10.65, − 5.27)	−5.52 (−7.92, − 3.11)
(2) Exercise or exercise + additional intervention	3	97	2.87 (1.42, 4.31)	−0.30 (−1.47, 0.87)
(3) Non-exercise intervention	6	190	1.14 (−3.37, 5.66)	−0.35 (−3.56, 2.86)
Length of yoga intervention				
(1) ≤ mean (58.9 hours)	16	728	−3.11 (−5.49, − 0.73)	−2.55 (−2.95, 2.15)
(2) > mean (58.9 hours)	6	215	−9.73 (−17.66, − 1.79)	−1.83 (3.59, − 0.07)

Types of yoga intervention: P: postures; B: breathing; M: meditation; 1 = P + M + B, 2 = any 2 of these or less; 3: (±P ± M ± B) + Other intervention.

Length of yoga intervention: 16 trials (12 studies) were categorized as being of short duration as they fell below the mean value across all studies of 58.9 hours; 6 trials (5 studies) were categorized as being of long duration.

*Significant effect of subgroup differences, *P* < 0.001.

## References

[1] Roger VL, Go AS, Lloyd-Jones DM (2012). On behalf of the American Heart Association statistics committee and stroke statistics subcommittee. Heart disease and stroke statistics-2012 update: a report from the American Heart Association. *Circulation*.

[2] Gillespie C, Kuklina EV, Briss PA, Blair NA, Hong Y (2011). Vital signs: prevalence, treatment, and control of hypertension, United States, 1999–2002 and 2005–2008. *Morbidity and Mortality Weekly Report*.

[3] Chobanian AV, Bakris GL, Black HR (2003). The Seventh Report of the Joint National Committee on Prevention, Detection, Evaluation, and Treatment of High Blood Pressure: the JNC 7 report. *Journal of the American Medical Association*.

[4] High blood pressure facts. http://www.cdc.gov/bloodpressure/facts.htm.

[5] Okonta NR (2012). Does yoga therapy reduce blood pressure in patients with hypertension?: an integrative review. *Holistic Nursing Practice*.

[6] Innes KE, Vincent HK (2007). The influence of yoga-based programs on risk profiles in adults with type 2 diabetes mellitus: a systematic review. *Evidence-Based Complementary and Alternative Medicine*.

[7] Innes KE, Bourguignon C, Taylor AG (2005). Risk indices associated with the insulin resistance syndrome, cardiovascular disease, and possible protection with yoga: a systematic review. *Journal of the American Board of Family Practice*.

[8] Walters JD (2002). *The Art and Science of Raja Yoga: Fourteen Steps to Higher Awareness*.

[9] Feuerstein G (2002). *The Yoga Tradition: Its History, Literature, Philosophy, and Practice*.

[10] Brown RP, Gerbarg PL (2005). Sudarshan Kriya yogic breathing in the treatment of stress, anxiety, and depression—part I: neurophysiologic model. *Journal of Alternative and Complementary Medicine*.

[11] Baldwin MC (1999). Psychological and physiological influences of hatha yoga training on healthy, exercising adults (yoga, stress, wellness). *Dissertation Abstracts International Section A*.

[12] Cowen VS, Adams TB (2005). Physical and perceptual benefits of yoga asana practice: results of a pilot study. *Journal of Bodywork and Movement Therapies*.

[13] Dvivedi J, Kaur H, Dvivedi S (2008). Effect of 1 week ’61-points relaxation training’ on cold pressor test induced stress in premenstrual syndrome. *Indian Journal of Physiology and Pharmacology*.

[14] Hutchinson S, Ernst E (2003). Yoga therapy for coronary heart disease: a systematic review. *Focus on Alternative and Complementary Therapies*.

[15] Raub JA (2002). Psychophysiologic effects of Hatha Yoga on musculoskeletal and cardiopulmonary function: a literature review. *Journal of Alternative and Complementary Medicine*.

[16] Jayasinghe SR (2004). Yoga in cardiac health (a review). *European Journal of Cardiovascular Prevention and Rehabilitation*.

[17] Bussing A, Michalsen A, Khalsa SB, Telles S, Sherman KJ (2012). Effects of yoga on mental and physical health: a short summary of reviews. *Evidence-Based Complementary and Alternative Medicine*.

[18] Anderson JW, Liu C, Kryscio RJ (2008). Blood pressure response to transcendental meditation: a meta-analysis. *American Journal of Hypertension*.

[19] Cade WT, Reeds DN, Mondy KE (2010). Yoga lifestyle intervention reduces blood pressure in HIV-infected adults with cardiovascular disease risk factors. *HIV Medicine*.

[20] Cohen BE, Chang AA, Grady D, Kanaya AM (2008). Restorative yoga in adults with metabolic syndrome: a randomized, controlled pilot trial. *Metabolic Syndrome and Related Disorders*.

[21] Cohen DL, Bloedon LT, Rothman RL (2011). Iyengar yoga versus enhanced usual care on blood pressure in patients with prehypertension to stage I hypertension: a randomized controlled trial. *Evidence-Based Complementary and Alternative Medicine*.

[22] McCaffrey R, Ruknui P, Hatthakit U, Kasetsomboon P (2005). The effects of yoga on hypertensive persons in Thailand. *Holistic Nursing Practice*.

[23] van Montfrans GA, Karemaker JM, Wieling W, Dunning AJ (1990). Relaxation therapy and continuous ambulatory blood pressure in mild hypertension: a controlled study. *British Medical Journal*.

[24] Murugesan R, Govindarajulu N, Bera TK (2000). Effect of selected yogic practices on the management of hypertension. *Indian Journal of Physiology and Pharmacology*.

[25] Patel C, North WRS (1975). Randomised controlled trial of yoga and bio feedback in management of hypertension. *The Lancet*.

[26] Saptharishi LG, Soudarssanane MB, Thiruselvakumar D (2009). Community-based randomized controlled trial of non-pharmacological interventions in prevention and control of hypertension among young adults. *Indian Journal of Community Medicine*.

[27] Subramanian H, Soudarssanane MB, Jayalakshmy R (2011). Non-pharmacological interventions in hypertension: a community-based cross-over randomized controlled trial. *Indian Journal of Community Medicine*.

[28] Deepa T, Sethu G, Thirrunavukkarasu N (2012). Effect of yoga and meditation on mild to moderate essential hypertensives. *Journal of Clinical and Diagnostic Research*.

[29] Hegde SV, Adhikari P, Kotian S, Pinto VJ, D'Souza S, D'Souza V (2011). Effect of 3-month yoga on oxidative stress in type 2 diabetes with or without complications. *Diabetes Care*.

[30] Jain S, Jain M, Sharma CS (2010). Effect of yoga and relaxation techniques on cardiovascular system. *Indian Journal of Physiology and Pharmacology*.

[31] Lakkireddy D, Atkins D, Pillarisetti J (2013). Effect of yoga on arrhythmia burden, anxiety, depression, and quality of life in paroxysmal atrial fibrillation: the YOGA My Heart Study. *Journal of the American College of Cardiology*.

[32] Mizuno J, Monteiro HL (2013). An assessment of a sequence of yoga exercises to patients with arterial hypertension. *Journal of Bodywork and Movement Therapies*.

[33] Niranjan M, Bhagyalakshmi K, Ganaraja B, Adhikari P, Bhat R (2009). Effects of yoga and supervised integrated exercise on heart rate variability and blood pressure in hypertensive patients. *Journal of Chinese Clinical Medicine*.

[34] Patel C (1975). 12-month follow up of yoga and bio feedback in the management of hypertension. *The Lancet*.

[35] Selvamurthy W, Sridharan K, Ray US (1998). A new physiological approach to control essential hypertension. *Indian Journal of Physiology and Pharmacology*.

[36] Gordon L, Morrison EY, McGrowder DA (2008). Changes in clinical and metabolic parameters after exercise therapy in patents with type 2 diabetes. *Archives of Medical Science*.

[37] (2011). *Review Manager (RevMan) [Computer Program]. Version 5.1*.

[38] Higgins JPT, Thompson SG (2002). Quantifying heterogeneity in a meta-analysis. *Statistics in Medicine*.

[39] Deeks J, Higgins JP, Higgins JP, Green S (2008). Analysing data and undertaking meta-analysis. *Cochrane Handbook for Systematic Reviews of Interventions*.

[40] Pullen PR (2009). *The Benefits of Yoga Therapy for Heart Failure Patients*.

[41] Chen KM, Fan JT, Wang HH, Wu SJ, Li CH, Lin HS (2010). Silver yoga exercises improved physical fitness of transitional frail elders. *Nursing Research*.

[42] Latha AU, Kaliappan KV (1991). Yoga, pranayama, thermal biofeedback techniques in the management of stress and high blood pressure. *Journal of Indian Psychology*.

[43] Haber D (1986). Health promotion to reduce blood pressure level among older blacks. *Gerontologist*.

[44] Haber D (1983). Yoga as a preventive health care program for white and black elders: an exploratory study. *International Journal of Aging and Human Development*.

[45] Mourya M, Mahajan AS, Singh NP, Jain AK (2009). Effect of slow- and fast-breathing exercises on autonomic functions in patients with essential hypertension. *Journal of Alternative and Complementary Medicine*.

[46] Khatri D, Mathur KC, Gahlot S, Jain S, Agrawal RP (2007). Effects of yoga and meditation on clinical and biochemical parameters of metabolic syndrome. *Diabetes Research and Clinical Practice*.

[47] Chung SC, Brooks MM, Rai M, Balk JL, Rai S (2012). Effect of sahaja yoga meditation on quality of life, anxiety, and blood pressure control. *Journal of Alternative and Complementary Medicine*.

[48] Pal A, Srivastava N, Tiwari S (2011). Effect of yogic practices on lipid profile and body fat composition in patients of coronary artery disease. *Complementary Therapies in Medicine*.

[49] Skoro-Kondza L, See Tai S, Gadelrab R, Drincevic D, Greenhalgh T (2009). Community based yoga classes for type 2 diabetes: an exploratory randomised controlled trial. *BMC Health Services Research*.

[50] Broota A, Varma R, Singh A (1995). Role of relaxation in hypertension. *Journal of the Indian Academy of Applied Psychology*.

[51] Chaudhary AK, Bhatnagar HN, Bhatnagar LK, Chaudhary K (1988). Comparative study of the effect of drugs and relaxation exercise (yoga shavasan) in hypertension. *The Journal of the Association of Physicians of India*.

[52] Yogendra J, Yogendra HJ, Ambardekar S (2004). Beneficial effects of Yoga lifestyle on reversibility of ischaemic heart disease: caring heart project of international board of Yoga. *Journal of Association of Physicians of India*.

[53] Sharma R, Gupta N, Bijlani RL (2008). Effect of yoga based lifestyle intervention on subjective well-being. *Indian Journal of Physiology and Pharmacology*.

